# Assessing self-forgiveness through the Enright Self-Forgiveness Inventory in the Spanish population: a validation study

**DOI:** 10.3389/fpsyg.2023.1179826

**Published:** 2023-08-17

**Authors:** Clara Molinero, Agata Kasprzak, Saray Bonete, Karla Gallo-Giunzioni

**Affiliations:** ^1^Faculty of Education and Psychology, Universidad Francisco de Vitoria, Madrid, Spain; ^2^Instituto del Perdón UFV, Madrid, Spain

**Keywords:** self-forgiveness, assessment, ESFI, validation, Spanish

## Abstract

**Introduction:**

Self-forgiveness has been a complex construct to define, which has resulted in a shortage of instruments that adequately measure it as a process. In Spain, until now there is only one validated instrument to measure self-forgiveness, for this reason the present study aims to validate the Enright Self-Forgiveness Inventory (ESFI).

**Method:**

A sample of 276 people (84 men, 192 women) aged from 18 to 25 years, completed the Enright Self-Forgiveness Inventory (ESFI) after its adaptation to Spanish, as well as the Enright Forgiveness Inventory-30 (EFI-30), the Narcissistic Personality Inventory (NPI), the Short form of Social Desirability Scale (M-C SDS), the Scale of psychological wellbeing (RYFF) and the Depression, Anxiety and Stress Scale-21 (DASS-21).

**Results:**

The Confirmatory Factor Analysis showed a good fit for the original six-factors structure (CFI = 0.93, TLI = 0.92, RMSEA = 0.063). The results showed good psychometric qualities (both validity and reliability) and association between self-forgiveness and social desirability, depression, anxiety, narcissistic traits, and purpose in life as expected theoretically.

**Discussion:**

The ESFI-30 shows good psychometric properties within the Spanish context and is an appropriate instrument for evaluating self-forgiveness for research and clinical intervention.

## Introduction

As people, we learn to interact with the world and with ourselves through interpersonal skills. It is widely recognised that positive relations are part of the wellbeing of human beings (Seligman, 2011). In the relational context, it is possible to feel hurt due to the behaviour of someone else or our own. Varied research (Worthington and Scherer, 2004; Prieto-Ursúa et al., 2012; Enright and Fitzgibbons, 2015) presents forgiveness as an ability which is trainable and contributes to the psychological wellbeing of individuals, facilitates personal restoration and relationships with others (Wade et al., 2014). This trainable essence of forgiveness means it can be developed as an alternative solution to interpersonal conflicts as it favours coexistence among people (D’Zurilla and Nezu, 2010; Bonete and Molinero, 2016; Vitz, 2018; García-Martín and Calero-García, 2019).

Self-forgiveness is not isolated from any other processes that influence the way in which human beings’ personalities are built, as learning or personal identity processes, which last a lifetime and might be especially at risk when dealing with each evolutionary task (Erikson, 1993; Dans-Álvarez-de-Sotomayor and Muñiz-Álvarez, 2021). In this process, we might get it wrong and make mistakes. The narrative of the person in these possible errors and the role played when harming occurs, can make self-forgiveness a key element in generating or avoiding clinical symptomatology (Van Vliet, 2008; Davis et al., 2015). Some studies on forgiveness indicate that, depending on the case, training this kind of forgiveness may bring about more significant changes in patients than traditional psychotherapy (Goldman and Wade, 2012).

The study of forgiveness has become more relevant in the last few decades, but only recently has it been of interest as a research topic in psychology. Particularly in Spain, scientific publications on this topic can be found from 2010 onward (Prieto-Ursúa and Echegoyen, 2015). This systematization appears to be linked to the growing rise experienced by Positive Psychology, which regards forgiveness as one of humans’ main strengths, given its benefits which are linked to happiness and personal wellbeing (Prieto-Ursúa and Echegoyen, 2015).

Before it was developed in psychology, other disciplines such as philosophy, religion and social sciences already referred to forgiveness as a positive behavior (Holmgren, 1998). Nonetheless, some authors postulated that forgiveness could be harmful to the health of anyone, it could perpetuate injustice or help an assailant to continue behaving incorrectly (Enright et al., 1992; Wohl and McLaughlin, 2014; Enright and Fitzgibbons, 2015; Song and Enright, 2021). This is what some studies have called a “dark side of forgiveness”; the situation where forgiveness becomes a conservation mechanism of unhealthy behaviours for anyone who commits an offence and their environment (McNulty, 2011; Wohl and Thompson, 2011). From then on, forgiveness has given rise to debate in some contexts.

Principles of forgiveness are found in the philosophy of Socrates, Plato and Aristotle. In philosophical terms, together with the four most well-known virtues (justice, courage, wisdom and temperance), magnanimity can also be considered. The last virtue mentioned refers to a fullness of the heart which indicates love and prepares a person to give to others beyond what is fair or deserved. In this respect, forgiveness is one way of being magnanimous, means carrying out a heroic virtue that gives the offender back the place in our heart that he had before the offence, even if this is not deserved (Enright and Fitzgibbons, 2015). In the words of Enright, forgiveness is “a response to an injustice which includes a reduction in resentment or rage towards the offender and the establishment of thoughts, feelings and positive behaviours towards other people” (Knutson et al., 2008).

As regards self-forgiveness, it can be stated that the only thing that changes is the person towards whom negative thoughts and feelings are reduced and positive ones are established. This way since forgiveness is a moral virtue it may therefore be used on oneself (Kim et al., 2021) and this may also include compassion, unconditional worth, moral love and generosity toward oneself (Kim and Enright, 2016). But defining self-forgiveness has not proved to be an easy task, as while some authors (Enright, 1996) suggested using the same definition for both interpersonal forgiveness and intrapersonal forgiveness, for other authors this would not be quite right (Cornish and Wade, 2015).

The origins of this definition of self-forgiveness are complemented by the studies of philosophers of Aristotelian tradition including the Kantian emphasis on good will and the subsequent work of authors such Holmgren and North, where forgiveness is justified by the intrinsic value of individuals, which means that people deserve our respect (Holmgren, 1998) or affection (North, 1987) beyond the acts committed; and this respect includes oneself when transgressing. Additionally, this approach highlights the importance of the content of the moral virtue and the practice of it within a specific context in order to become a morally virtuous person (Enright and Fitzgibbons, 2015; Kim et al., 2021). As in interpersonal forgiveness, this does not imply a justifying or re-establishing of the processes concerning the offence, but it does signify an awareness of the person’s own value regardless of the act committed. Consequently, according to this definition of self-forgiveness, it is important to recognise the injustice under the wrongdoing or the offence towards oneself which generated emotions such as guilt and shame, otherwise the presence of these emotions themselves will not give rise to the process of self-forgiveness (Kim et al., 2021).

One of the consequences associated with the difficulty of defining self-forgiveness is the scarcity of instruments that really measure forgiveness as a process and not only as a final result. The following stand out among these instruments: The State Self-Forgiveness Scale-SSFS (Wahkinney, 2002); the Self-Forgiveness Single Item (Wohl et al., 2008); the Heartland Forgiveness Scale (Thompson et al., 2005); the Differentiated Process Scale of Self-Forgiveness (Woodyatt and Wenzel, 2013); and the Enright Self-Forgiveness Inventory (ESFI) (Kim et al., 2021). On the other hand, the validation of the ESFI, it would not only be providing a measure that analyzes the forgiveness process, what self-forgiveness is and what it is not. Furthermore, it would be the first evaluation instrument available to the Spanish-speaking population whose essence is to understand forgiveness as a moral virtue.

In Spain and Spanish-speaking countries, the most commonly used instrument to measure self-forgiveness is the Heartland Forgiveness Scale (HFS), an instrument which has recently been validated in the Spanish population (Gallo-Giunzioni et al., 2021). It is worth noting that up to now, it has been the only measure to be translated into and adapted to Spanish which has been made available, even though the authors of the validation reported that considerable adjustments must be made to the number of items that make up the scale in order to improve the way it works in our population.

The aim of this study is to validate a second instrument to evaluate self-forgiveness in the Spanish population in order to widen the variety of measures, so as to be able to continue studying the self-forgiveness process in more detail and foster support in the process of shaping personal identity and maturity (Worthington et al., 2007). The intention is to adapt to Spanish population the Enright Self-Forgiveness Inventory (ESFI), which was created and validated by Robert Enright and his collaborators (Kim et al., 2021). Made up of 30 items and six subscales, it provides information on positive and negative affect towards the self, positive and negative thoughts towards the self, positive and negative behaviour towards the self and it also has another scale of pseudo self-forgiveness. It was proposed as a clinical tool to identify who is ready to train the willingness of self-forgiveness, who might benefit from forgiveness interventions as well as to document their progress towards self-forgiveness programs. It is hoped that this instrument, which has shown good results in its use with ordinary people and in clinical samples (Kim and Enright, 2016; Martinčeková and Enright, 2020), will also be valid for use in Spanish samples.

## Materials and methods

### Participants

From an initial sample of 396 participants, the valid responses of 276 people are analysed: 84 men (30.4%) and 192 women (69.6%) residents of the Autonomous Region of Madrid with postgraduate/doctorate studies (10.9%), bachelor’s degree (55.1%), vocational training (6.5%), basic education (26.4%), no education (1.1%). Ages range between 18 and 64 years, and 93.5% of participants are aged between 18 and 25 years. All volunteers took part based on non-probability and snowball sampling. Participation in the research was disseminated on the university campus by means of posters and lecturers and on social networks (for example, on LinkedIn). The inclusion criteria were as follows: (1) accept voluntary participation in the study, (2) be aged between 18 and 75 years, (3) not have any severe psychopathological diagnosis.

### Measures

Enright Self-Forgiveness Inventory (ESFI) (Kim et al., 2021). The instrument has 30 items in a Likert scale with six response options, divided into six subscales. The scale measures self-forgiveness in the context of a specific offence. The authors have reported appropriate internal consistency rates for the affective scale (*α* = 0.97), for the behavioural scale (*α* = 0.85) and for the cognitive scale (*α* = 0.94). Within this instrument, we have also included the five pseudo self-forgiveness items created by Enright as a measure of the quality of the forgiveness process.

Enright Forgiveness Inventory (EFI) (Enright et al., 2022). The inventory has three subscales: affective, behavioural and cognitive; each one is made up of 10 items (5 written positively and 5 written negatively) giving rise to the subdimensions of Positive Affect (PA), Negative Affect (NA), Positive Behaviour (PB), Negative Behaviour (NB), Positive Cognition (PC) and Negative Cognition (NC). Each item is responded to in accordance with a 6-point Likert-type scale, where the higher the score, the higher the forgiveness with regard to a specific offence. In relation to consistency rates, the authors report good psychometric properties of the instrument: *α* = 0.98 for the affective scale, *α* = 0.97 for the behavioural scale and *α* = 0.96 for the cognitive scale. In this research, the following was obtained: *α* = 0.75 for the affective scale, *α* = 0.77 for the behavioural scale and *α* = 0.73 for the cognitive scale.

Narcissistic Personality Inventory (NPI) (Raskin and Terry, 1988); adaptation to Spanish (Martinčeková and Enright, 2020). Reduced version of 40 items, which is designed to measure the degree to which people differ in narcissism as a personality trait. It is made up of 7 subscales: (a) authority, (b) exhibitionism, (c) superiority, (d) entitlement, (e) exploitativeness, (f) self-sufficiency, and (g) vanity. Each item has two response options where the participant must choose with which one, they are best identified. Some examples of the items found on the scale are: “I am more capable than other people; There is a lot that I can learn from other people” “I am much like everybody else; I am an extraordinary person.” García Garduño and Cortés Sotrés (1998) reported an appropriate level of reliability of the total instrument (*α =* 0.72) for Spanish samples. In this research, *α* = 0.64 was obtained for the whole test.

Short Form of Social Desirability Scale (M-C SDS) (Crowne and Marlowe, 1960); Spanish adaptation (Gutiérrez et al., 2016). Made up of 18 items where the subjects respond to a series of hypothetical statements about themselves, considering the response as true or false. This scale is used to evaluate social desirability, bearing in mind that a higher score indicates a higher social desirability, understood as response bias or defensiveness. The Spanish version obtains appropriate internal consistency rates (*α =* 0.76). In this research, *α* = 0.40 was obtained.

Scale of Psychological Wellbeing (RYFF) (Van Dierendonck et al., 2008); adaptation to Spanish samples (Díaz et al., 2006). This instrument is designed to measure psychological wellbeing using 29 items with a 6-point Likert-type response format (1 = strongly disagree up to 6 = strongly agree). It is made up of 6 subscales: self-acceptance, positive relations, autonomy, environmental mastery, purpose in life and personal growth. The authors have reported appropriate internal consistency levels for each one of the subscales, recording over 0.70 (Crowne and Marlowe, 1960). Given that the interest in this research is the overall psychological wellbeing conceived, the total sum of the items will be used to obtain an overall score. In this research, an internal consistency rate of 0.87 was obtained for the whole test.

Depression, Anxiety and Stress Scale-21 (DASS-21) (Lovibond and Lovibond, 1995). It is made up of 21 items with a 4-point Likert-type response format divided into three subscales: (a) Depression: evaluates various symptoms of depression such as dysphoria, devaluation of life, hopelessness, self-deprecation, lack of interest or involvement, anhedonia, and inertia. (b) Anxiety: measures worries, somatic and subjective symptoms of fear, arousal, muscle effects, situational anxiety and subjective experience of anxious affect. (c) Stress: evaluates arousal and tension, difficulty relaxing, nervousness and being easily upset, agitated or irritated. Its authors reported appropriate internal consistency values, with Cronbach’s *α* of 0.91 for the depression scale, 0.84 for the anxiety scale and 0.90 for the stress scale (Bados et al., 2005). In this research, the following was obtained: *α* = 0.88 for the depression scale, *α* = 0.84 for the anxiety scale and *α*= 0.83 for the stress scale.

### Procedure

Once permission had been given by the original authors for the translation, adaptation and validation of the scale, the process of translating and reverse translating into English was carried out as suggested by regular psychometric recommendations (Muñiz et al., 2013). This was subject to the approval of the Ethics Committee of the Francisco de Vitoria University. The participants filled out an anonymous questionnaire online via Qualtrics which started with informed consent. They were able to access the questionnaire using their mobile devices or computers. Tests were presented in a counterbalanced order, with the ESFI test always at first.

### Statistical analyses

This is an ex-post facto study which uses non-probability sampling to collect the characteristics of an adult Spanish population sample with respect to the willingness of self-forgiveness and some associated psychological variables.

At a preliminary descriptive level, the characteristics of the sample were reviewed in all self-forgiveness (ESFI) scores. A Confirmatory Factor Analysis was conducted in order to evaluate internal validity, followed by a reliability analysis for all the ESFI subscales. Furthermore, concurrent validity of the ESFI was reviewed based on the correlations between the variables studied.

The statistical processing of the data collected will be carried out using the statistical package SPSS 25.0 and AMOS 26.

## Results

### Preliminary analysis of the ESFI items

The descriptive analysis of the ESFI items (mean, standard deviation, skewness and kurtosis of each one) (Supplementary Table S1) reported similar distributions of the response alternatives and appropriate skewness and kurtosis values (below |1.5|). The items’ mean (considering ESFI is a 30 items 6-point Likert-type scale) varies between 1.93 (item 21) and 4.50 (item 30). Standard deviations range between 1.23 (item 21) and 1.76 (item 11), therefore it can be stated that there is appropriate variability in the scores. Below, Means and Standard Deviation for Subscales and Total Scale scores are shown; they may be used as pilot normative data for similar populations (Supplementary Table S2).

### Internal validity: confirmatory factor analysis

**Figure 1:**
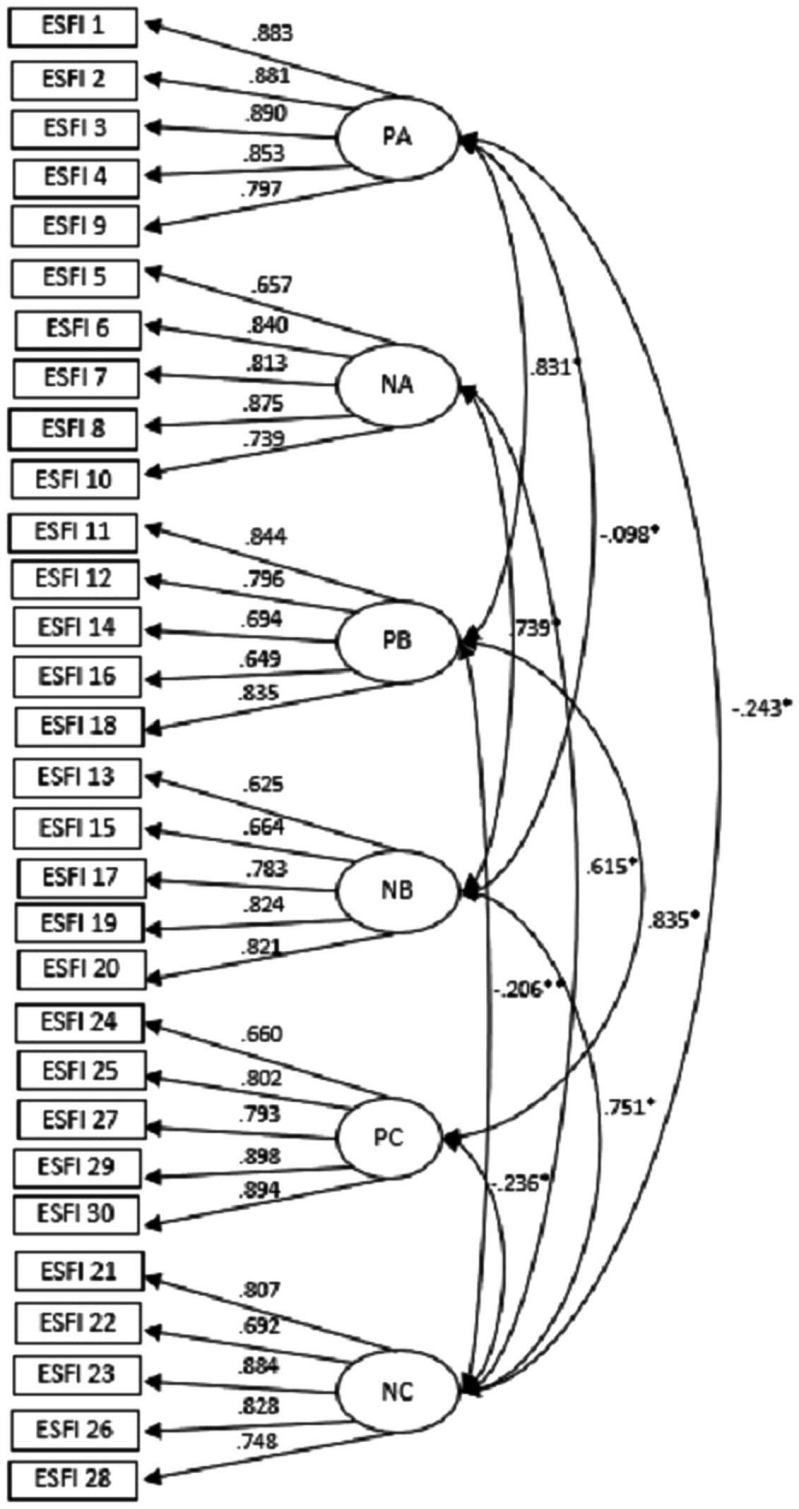
Model. *s < 0.0001; **s < 0.003.

In order to check the ESFI-30 structure proposed by Enright (Kim et al., 2021) in a Spanish sample, a Confirmatory Factor Analysis was conducted on six correlated factors according to the six original ESFI-30 subscales using the Maximum Likelihood Method. The results of the analysis indicated a good fit of the model. Firstly, an RMSEA value of 0.063 was obtained, which indicates a good fit as it is below 0.07 (Hooper et al., 2008). As for the SRMR index, a value of 0.0611 was obtained, with appropriate values considered to be between 0.05 and 0.08. The CFI and TLI show a value higher than 0.90, which coincides with the values recommended by Marsh et al. (2004) and Markland (2007). As can be seen in Figure 1, the range of factor loadings for the model varies between 0.625 (item 13) and 0.898 (item 29) (Supplementary Table S3).

### Reliability of ESFI-30

An internal consistency analysis has been conducted for the entire test and for each scale using Cronbach’s alpha (Supplementary Table S4). Cronbach’s alpha was 0.90 for the whole test, alphas values for each subscale are shown below (Supplementary Table S4).

### Convergent validity of ESFI-30 and its subscales

The convergent validity was examined between ESFI-30 and social desirability evaluated using M-C SDS; anxiety depression and stress using DASS-21; narcissistic traits evaluated using NPI (Total Scores and each subscale) and psychological wellbeing using the RYFF Scale of Psychological Wellbeing (Supplementary Table S5).

### Criterion validity of ESFI-30

The criterion validity of ESFI-30 and its scales was also reviewed using Pearson correlation with the following EFI-30 scales: Positive Affect, Negative Affect, Positive Behaviour, Negative Behaviour, Positive Cognition and Negative Cognition (Supplementary Table S6).

## Discussion

The aim of this study was to adapt the Self-Forgiveness Scale, developed by Robert Enright, to a Spanish sample and review its psychometric quality. This scale was supported due to the richness of the theoretical conceptualization of the forgiveness construct of this author (Wade et al., 2014; Enright and Fitzgibbons, 2015) and the positive effects reported by the various studies which examine the forgiveness intervention based on his proposal (Zhang et al., 2014; Ji et al., 2016a,b; Kim et al., 2022).

The reliability analyses conducted showed a sound internal consistency and, after all the analyses done, descriptive means and standard deviations might be used as a preliminary normative data for further comparisons with other Spanish samples.

Internal validity reviewed by the CFA showed a good fit for the model, similar to the theoretical model Enright had proposed in the USA version of the ESFI (Kim et al., 2021). Factors (Positive and Negative Affect, Cognition and Behaviour) appeared correlated in the expected ways: positives together and negatives together, and with the opposite direction between them. It was noticed that negative subscales relations appeared stronger than relations between positives subscales. It seems that thoughts against the offender (one’s own in the case of self-forgiveness) go stronger together with negative affects and behaviours, and positive ones take a little more effort to converge. As stated before, forgiveness (both interpersonal and self-forgiveness) is a virtue, and takes work, training and practice; while resentment and this kind of feelings following hurt are hard to work out (Levy et al., 2021).

One of the potentialities to note from this study is that we obtain a very good psychometric quality following the use of the instrument translated into Spanish without having to make adjustments to the number of items that may affect the validity of the aforementioned. Previous research with the Heartland Forgiveness Scale (HFS) by Thompson et al. (2005) concluded that, in order to be used in the Spanish population and maintain the factorial structure proposed by the original authors, it must be adapted to an abbreviated version with eight items (Gallo-Giunzioni et al., 2021). Theoretical implications of this structure and content stability are not only the transcultural validity; it is also evidence that ESFI approaches to the human process of self-forgiveness in a better way. This more accurate approach broadens practical possibilities, because considering self-forgiveness as a moral virtue implies it is trainable, and also has the advantage of including not only affects and cognitions over the self but also behaviours (Kim et al., 2021).

Most previous studies that examined the effectiveness of self-forgiveness interventions (Griffin et al., 2015; Bell et al., 2017) used the Heartland Forgiveness Scale (HFS) by Thompson et al. (2005) as an instrument to assess change of the willingness to self-forgiveness. Since HFS has some difficulties in Spanish samples, having an additional instrument is a boost to validate self-forgiveness training programmes in the Spanish population.

Concurrent validity results (correlations between all the measures) went beyond proving psychometrical quality. These correlations showed very interesting relations between variables. First, social desirability showed to be independent of the ESFI total score and negative correlated (significant but weak) to negative subscales. This shows how participants recognize that negative affect, behaviour, and cognitions are not desirable; but total ESFI scores resisted well to social desirability.

Second, depression and anxiety symptoms showed to be linked to self-forgiveness, especially to negative subscales of affect, behaviour, and cognition. Numerous studies have already analysed this link, more commonly on interpersonal forgiveness (Gao et al., 2022); now there is one more evidence about self-forgiveness too (Costa et al., 2021; Kim et al., 2021).

About narcissism traits, it was a surprising outcome how higher scores on narcissism had positive significant but small correlations with negative affect, negative behaviour and negative cognition toward oneself. This result opens a gate to further research about how narcissism might simulate high acceptance of oneself wrongdoing, although some pain after offending might be underlying. When looking at the subscales, these correlations are higher in exhibitionism, entitlement and exploitativeness. These subscales have proved to be linked in previous research (Hill and Yousey, 2017), they highlight an exceptional expectation over oneself and self-exigency, hence it might be showing difficulties to accept one’s own mistakes. Another result that points in the same direction is the correlation between self-forgiveness and self-acceptance; seems that leading with one’s own wrongdoing requires some humbleness (Fisher, 2020). Some other facilitators of self-forgiveness were environmental mastery, purpose in life and personal growth. Perhaps further studies add some of those in order enhance self-forgiveness development (Van Dyke and Elias, 2007; Lyons et al., 2011). This study explores relations between self-forgiveness and personality traits (Kim et al., 2021), that could be widely developed in the future due to its relevant implications in psychotherapy.

Finally, another interesting result is correlation between interpersonal forgiveness (EFI-30) and self-forgiveness (ESFI-30). Association between scores was small at total scores, but it emerged when subscales were analysed separately. It seems that independence between all types of forgiveness cannot be assumed, as has been shown in other studies: the skill of forgiving can be developed by training and its benefits reach others and oneself together (Fincham and May, 2021).

In terms of limitations observed in this study, we recognise that the sample size is small, and this may affect the extent into which these results can be generalized; as well as the heterogeneity of men and women. For future research, we suggest that these limitations should not only be considered and corrected, but also that the study should be extended to contexts that go beyond the university on which a great deal of the research has been focused (Liao and Wei, 2015; Martinčeková and Enright, 2020). The next step is to examine the functioning of the scale in a clinical population, social minorities, etc., for whom working with this questionnaire may also be used as a measure for change following an intervention.

We believe that the value of this research lies in the scarcity of translated and validated instruments in a Spanish sample. Having a self-forgiveness measure which is one of the most widely used in research worldwide, and which works correctly in our population is a breakthrough in the study of this construct in our country, given the interesting implications in fields such as clinical intervention and psychotherapy. In this context, Enright (1996) proposes self-forgiveness as the desire to abandon self-resentment in the face of one’s own acknowledged objective wrong, thus fostering generosity, compassion and love towards oneself. In this respect, the results of various studies (Hall and Fincham, 2005; Thompson et al., 2005; Fisher and Exline, 2006; Liao and Wei, 2015) suggested that the lack of forgiveness in the face of one’s own wrongs predicts low self-esteem and high levels of guilt and relates to higher levels of psychopathology (Van Dyke and Elias, 2007). Some studies indicate that self-forgiveness training is even more closely related to the relief of symptomatology than interpersonal forgiveness (Worthington et al., 2007; Gençoğlu et al., 2018) with beneficial results in the treatment of alcoholism (Scherer et al., 2011) and other problems.

## Data availability statement

The raw data supporting the conclusions of this article will be made available by the authors, without undue reservation.

## Ethics statement

The studies involving humans were approved by Ethics Committee of Universidad Francisco de Vitoria (protocol code 52/2021 and date of approval 3 November 2021). The studies were conducted in accordance with the local legislation and institutional requirements. The participants provided their written informed consent to participate in this study.

## Author contributions

CM, AK, SB, and KG-G: conceptualization and design, investigation, writing—original draft, and writing—review and editing. SB and CM: methodology and sample recruitment. CM: provide approval for publication of the content. AK: data curation and formal analysis. All authors contributed to the article and approved the submitted version.

## Funding

This research is one of the works carried out at the Instituto del Perdón UFV. The funds to finance it arise from this institute.

## Conflict of interest

The authors declare that the research was conducted in the absence of any commercial or financial relationships that could be construed as a potential conflict of interest.

## Publisher’s note

All claims expressed in this article are solely those of the authors and do not necessarily represent those of their affiliated organizations, or those of the publisher, the editors and the reviewers. Any product that may be evaluated in this article, or claim that may be made by its manufacturer, is not guaranteed or endorsed by the publisher.
