# Supplementary material for: Assessing self-forgiveness through the Enright Self-Forgiveness Inventory in the Spanish population: a validation study
Source: Front Psychol. 2023 Aug 17;14:1179826. doi: 10.3389/fpsyg.2023.1179826 (PMC10469898; doi:10.3389/fpsyg.2023.1179826)
Supplement: Supplementary file 1 [file Data_Sheet_1.docx]

Supplementary Material

# Supplementary Figures and Tables

**Table 1**

*Descriptive data of the items*

| Item |  | Mean | SD | Skewness | Kurtosis |
| --- | --- | --- | --- | --- | --- |
| ESFI_1 |  | 3.77 | 1.53 | -0.26 | -0.94 |
| ESFI_2 |  | 3.87 | 1.60 | -0.39 | -1.04 |
| ESFI_3 |  | 3.82 | 1.51 | -0.25 | -0.97 |
| ESFI_3 |  | 3.82 | 1.51 | -0.25 | -0.97 |
| ESFI_4 |  | 3.72 | 1.57 | -0.16 | -1.10 |
| ESFI_5 |  | 2.71 | 1.36 | 0.56 | -0.56 |
| ESFI_6 |  | 2.62 | 1.44 | 0.68 | -0.43 |
| ESFI_7 |  | 2.55 | 1.45 | 0.82 | -0.22 |
| ESFI_8 |  | 2.49 | 1.33 | 0.75 | -0.19 |
| ESFI_9 |  | 3.78 | 1.51 | -0.28 | -1.02 |
| ESFI_10 |  | 2.34 | 1.38 | 0.98 | 0.15 |
| ESFI_11 |  | 4.47 | 1.76 | -0.89 | -0.63 |
| ESFI_12 |  | 3.97 | 1.61 | -0.30 | -1.15 |
| ESFI_13 |  | 3.08 | 1.49 | 0.28 | -0.88 |
| ESFI_14 |  | 3.73 | 1.57 | -0.07 | -1.16 |
| ESFI_15 |  | 2.77 | 1.46 | 0.54 | -0.68 |
| ESFI_16 |  | 3.63 | 1.54 | 0.01 | -1.08 |
| ESFI_17 |  | 2.88 | 1.44 | 0.44 | -0.69 |
| ESFI_18 |  | 4.05 | 1.47 | -0.42 | -0.83 |
| ESFI_19 |  | 2.62 | 1.45 | 0.66 | -0.57 |
| ESFI_20 |  | 2.28 | 1.45 | 1.06 | 0.19 |
| ESFI_21 |  | 1.93 | 1.23 | 1.32 | 1.04 |
| ESFI_22 |  | 2.07 | 1.33 | 1.27 | 0.83 |
| ESFI_23 |  | 1.98 | 1.28 | 1.31 | 0.90 |
| ESFI_24 |  | 3.68 | 1.46 | -0.21 | -0.92 |
| ESFI_25 |  | 4.23 | 1.65 | -0.54 | -0.98 |
| ESFI_26 |  | 2.03 | 1.32 | 1.28 | 0.81 |
| ESFI_27 |  | 4.03 | 1.48 | -0.36 | -0.79 |
| ESFI_28 |  | 2.30 | 1.35 | 0.85 | -0.14 |
| ESFI_29 |  | 4.21 | 1.58 | -0.64 | -0.72 |
| ESFI_30 |  | 4.50 | 1.58 | -0.82 | -0.56 |

| **Table 2** | | |
| --- | --- | --- |
| *Descriptive data by ESFI dimensions. Pilot normative ESFI scores.* | | |
|  | Mean | SD |
| Positive Affect (PA) | 18.95 | 6.86 |
| Negative Affect (NA) | 12.71 | 5.78 |
| Positive Behaviour (PB) | 19.85 | 6.52 |
| Negative Behaviour (NB) | 13.62 | 5.85 |
| Positive Cognition (PC) | 20.64 | 6.60 |
| Negative Cognition (NC) | 10.31 | 5.43 |
| ESFI Total Score | 94.97 | 23.13 |

**Table 3**

*Fit indexes of the model*

|  |  |  |  |  |  |  | IC 90% of the RMSEA | |  |  |
| --- | --- | --- | --- | --- | --- | --- | --- | --- | --- | --- |
| χ² | gl | p | CFI | TLI | SRMR | RMSEA | Lower | Higher | AIC | BIC |
| 817 | 390 | < .001 | .930 | .922 | .0611 | .0630 | .0569 | .0690 | 24333 | 24713 |

**Table 4.**

*Cronbach’s alpha*

|  | Cronbach's alpha |
| --- | --- |
| PA Scale | .93 |
| NA Scale | .89 |
| PB Scale | .88 |
| NB Scale | .86 |
| PC Scale | .91 |
| NC Scale | .89 |

**Table 5**

Correlations between ESFI-30 (Total and Subscales scores) and MC-SDS, DASS, NPI and Ryff Scales

|  |  |  |  | ESFI-30 Scales | | | | |
| --- | --- | --- | --- | --- | --- | --- | --- | --- |
|  | | ESFI-30-TS | Positive Affect (PA) | Negative Affect (NA) | Positive Behaviour (PB) | Negative Behaviour (NB) | Positive Cognition (PC) | Negative Cognition (NC) |
|  | MC-SDS-TS | -.071 | .117 | -.206^**^ | .087 | -.232^**^ | .118 | -.239^**^ |
|  | DASS-TS | -.102 | .259 | .342 | -.348 | -.087 | .204 | .436^*^ |
| DASS Scales | Depression | .097 | .260^**^ | .368^**^ | -.188^**^ | .418^**^ | -.203^**^ | .450^**^ |
|  | Anxiety | .095 | -.196^**^ | .329^**^ | -.183^**^ | .409^**^ | -.209^**^ | .416^**^ |
|  | Stress | .099 | -.189^**^ | .308^**^ | -.139^*^ | .335^**^ | -.115 | .317^**^ |
|  | NPI-TS | .147^*^ | -.093 | .269^**^ | -.104 | .284^**^ | -.066 | .341^**^ |
| NPI Scales | Authority | .015 | .011 | -.025 | -.013 | -.006 | -.010 | .019 |
|  | Exhibitionism | -.023 | -.134^*^ | .280^*^ | -.194^**^ | .164^**^ | -.227^**^ | .228^**^ |
|  | Superiority | .076 | .022 | .041 | -.024 | .088 | .004 | .126^*^ |
|  | Entitlement | .004 | -.213^**^ | .225^**^ | -.230^**^ | .263^**^ | -.274^**^ | .328^**^ |
|  | Exploitativeness | -.046 | -.149^*^ | .085 | -.203^**^ | .137^*^ | -.192^**^ | .127^*^ |
|  | Self-sufficiency | .045 | .109 | -.083 | .033 | -.059 | .068 | -.033 |
|  | Vanity | .071 | .172^**^ | -.174^**^ | .164^**^ | -.093 | .142^*^ | -.072 |
|  | RYFF-TS | .293^**^ | .446^**^ | -.170^**^ | .424^**^ | -.155^*^ | .478^**^ | -.223^**^ |
| Ryff Scales | Self-acceptance | .312^**^ | .432^**^ | -.185^**^ | .479^**^ | -.129^*^ | .526^**^ | -.215^**^ |
|  | Positive relations | .188^**^ | .327^**^ | -.155^*^ | .298^**^ | -.134^*^ | .354^**^ | -.207^**^ |
|  | Autonomy | .098 | .248^**^ | -.177^**^ | .197^**^ | -.196^**^ | .247^**^ | -.158^**^ |
|  | Environmental mastery | .298^**^ | .462^**^ | -.129^*^ | .416^**^ | -.155^**^ | .455^**^ | -.216^**^ |
|  | Purpose in life | .352^**^ | .468^**^ | -.124^*^ | .474^**^ | -.107 | .507^**^ | -.186^**^ |
|  | Personal growth | .310^**^ | .421^**^ | -.110 | .385^**^ | -.076 | .446^**^ | -.188^**^ |

*Note: *s<.05; ** s<.01; TS: Total score*

**Table 6**

Correlations between ESFI-30 and EFI-30

|  | |  | ESFI-30 Scales | | | | | |
| --- | --- | --- | --- | --- | --- | --- | --- | --- |
|  | | ESFI-30-TS | Positive Affect (PA) | Negative Affect (NA) | Positive Behaviour (PB) | Negative Behaviour (NB) | Positive Cognition (PC) | Negative Cognition (NC) |
|  | EFI-30-TS | .132^*^ | .350^**^ | -.230^**^ | .306^**^ | -.189^**^ | .355^**^ | -.322^**^ |
| EFI-30  Scales | Positive Affect (PA) | .460^**^ | .487^**^ | .049 | .429^**^ | .107 | .493^**^ | -.002 |
|  | Negative Affect (NA) | .388^**^ | .052 | .371^**^ | .134^*^ | .408^**^ | .141^*^ | .444^**^ |
|  | Positive Behaviour (PB) | .497^**^ | .491^**^ | .107 | .442^**^ | .144^*^ | .492^**^ | .012 |
|  | Negative Behaviour (NB) | .403^**^ | .044 | .413^**^ | .125^*^ | .419^**^ | .176^**^ | .425^**^ |
|  | Positive Cognition (PC) | .554^**^ | .552^**^ | .099 | .538^**^ | .109 | .592^**^ | -.054 |
|  | Negative Cognition (NC) | .315^**^ | -.024 | .390^**^ | .011 | .419^**^ | -.011 | .533^**^ |

*Note: *s<.05; ** s<.01; TS-Total score*
